# Bibliometric and visualization analysis of arginine deiminase research from 2006 to 2025: trends, collaboration networks and emerging frontiers

**DOI:** 10.3389/fimmu.2026.1784910

**Published:** 2026-07-22

**Authors:** Songtao Jiang, Lebin Gan, Qiang Wang, Guozheng Cao, Zhuang Zhang, Feifei Jin, Jing Zhou, Panpan Chang, Tianbing Wang, Jingjing Ye

**Affiliations:** Trauma Treatment Center, Peking University People’s Hospital; Key Laboratory of Trauma Treatment and Neural Regeneration (Peking University) Ministry of Education; National Center for Trauma Medicine, Beijing, China

**Keywords:** autoimmune and immune-related diseases, bibliometric analysis, citrullination, PADs, visualization analysis

## Abstract

**Background:**

Peptidylarginine Deiminase/Protein Arginine Deiminase (PAD/PADI) are a family of enzymes that catalyze protein citrullination, converting arginine residues to citrulline residues in a calcium-dependent manner. The net loss of positive charge of protein citrullination results in change of conformation, including probable formation of neoepitopes, and interactions with substances like DNA, which contributes to physiologic and pathophysiologic processes such as regulation of gene expression, Neutrophil Extracellular Traps (NETs), Rheumatoid Arthritis (RA), and Multiple Sclerosis (MS). To date, studies of PADs pan- and selective inhibitors have been deepening. This bibliometric analysis tries to give a comprehensive conclusion on research progress of peptidylarginine deiminases (PAD) and PAD inhibitors.

**Methods:**

In this study, we aggregated publications using a dual-database strategy (Web of Science and PubMed) from January 1, 2006, to December 5, 2025, with a specific focus on the arginine deiminase research. Utilizing bibliometric methods, the data underwent processing to facilitate visual analysis of various aspects, including countries, institutions, authors, co-citations, keywords, references, gene characteristics, and diseases.

**Results:**

Over the past two decades, the number of publications on the PAD family has increased continuously, with the United States contributing the largest number of publications. The University of Massachusetts emerged as the most prolific institution with 73 publications, while Paul R. Thompson was identified as the leading author in this field. Frontiers in Immunology published the highest number of PAD-related articles. High-frequency keywords included “rheumatoid arthritis”, “neutrophil extracellular traps”, “citrullination”, “PADI”, and “arginine deiminase”. Recent trends, identified through strong citation bursts from 2022 to 2025, include neutrophil extracellular traps, PADI4, and the tumor microenvironment. The high-frequency genes in the PADs field include PADI4, PADI2, TP53, and MPO. Additionally, pathways such as Lipid and atherosclerosis and viral infection-related pathways are significantly enriched in this research domain.

**Conclusions:**

The research maps the evolving landscape of PAD research over the past two decades. However, cooperation between institutions and countries remains limited, although trends indicate increasing cooperation. This study identifies major research hotspots and evolving trends within the PAD field, helping to guide future research directions and providing an objective, data-driven reference for tracking new frontiers in the field, rather than drawing biological conclusions.

## 1 Introduction

The peptidyl arginine deiminase (PAD/PADI) family is a group of calcium-dependent isoenzymes which mediates citrullination, catalyzes the conversion of arginine residues to citrulline residues of proteins (1, 2). PAD-mediated protein citrullination was involved in the process of cell death, influenced the formation of neutrophil extracellular traps (NETs) (3–5), inflammatory cytokine secretion, and the production of anti-citrulline protein antibodies (ACPA) involved in autoimmune diseases (6–8). Therefore, the PAD family is associated with the development of inflammatory autoimmune diseases and cancer, reproductive development, and other related diseases (1, 9).

The PAD enzyme family has been identified with 5 isoenzymes, including PAD1, PAD2, PAD3, PAD4, and PAD6, which are widely distributed in various tissues and cells. In accordance with standard nomenclature, the genes encoding these enzymes are referred to as PADI1–4 and PADI6, and are located on chromosome 1p36, while the corresponding functional proteins are designated as PAD1–4 and PAD6 (10, 11). PAD needs a certain concentration of Ca^2+^ in order to carry out its activity, catalyzing the conversion of arginine to protein to citrulline. Under normal physiological conditions, the concentration of intracellular Ca^2+^ is much lower than the concentration of PAD activation (12). When apoptosis or necrosis occurs under certain pathological conditions, such as inflammation, stress, hypoxia, etc., a large number of Ca^2+^ flow in, activating PAD, and PAD can also be secreted into the extracellular (13). This enables citrullination of proteins both inside and outside the cell, including filaggrin, keratin, histone, antithrombin, vimentin, fibrinogen, and fibrin, et al.

Previous literature has studied the function of PAD in physiological and pathological conditions, the pathways involved, and the relationship with disease. However, navigating through the vast literature without a proper strategy can be difficult, making it challenging to extract relevant information. In this study, we aimed to conduct quantitative literature research and visual analysis of the research development of the PAD enzyme family over the past two decades using bibliometric analysis. We aim to highlight hotspots, great teams, prolific research institutions, and advanced achievements in PAD enzyme study. We aim to provide new insights for applied and basic research in this field, increasing collaboration and communication. This research serves as a valuable resource for both experienced professionals and newcomers, allowing them to comprehensively assess the field, identify new areas of interest, and use a visual approach to develop informed strategies for future research. Such an approach significantly increases researchers’ efficiency and effectiveness. To the best of our knowledge, no previous bibliometric surveys have specifically focused on this topic.

## 2 Materials and methods

### 2.1 Data collection and retrieval methods

A comprehensive dual-database search strategy was implemented using the Web of Science Core Collection (WoSCC) and PubMed to ensure maximum data coverage. Data retrieval was conducted on December 5, 2025, spanning the period from January 1, 2006, to December 5, 2025. The search queries targeted “Padi1” or “Padi2” or “Padi3” or “Padi4” or “Padi6” or “Arginine Deiminase”. The initial search yielded 3,598 articles from WoSCC and 1,875 from PubMed. Inclusion criteria were restricted to English-language “Articles” and “Reviews”. After removing duplicates (1684 duplicate records) and irrelevant entries (391 records, e.g., meeting abstracts, editorial materials, early access, letters, corrections, book chapters), the remaining articles were merged, resulting in a final corpus of 3,398 publications. This data was used for quantitative and visualization-based bibliometric analyses of countries/regions, institutions, authors, journals, fields, references, keywords, genes, and diseases.

### 2.2 Bibliometric methods and visual analysis

Bibliometric analysis of the retrieved literature was collected using various tools and software, including VOSviewer 1.6.18 (Centre for Science and Technology Studies, Leiden University, The Netherlands), Citespace 6.3.R1 (Chaomei Chen, China), Pajek 64 5.16 (University of Ljubljana, Slovenia), Scimago Graphica 1.0.35 (https://www.graphica.app/, USA), R Package (Clusterprofiler, enrichplot, ggplot2, ComplexHeatmap 2.16.0, circlize 0.4.15), STRING (http://string-db.org) online platform, Cytoscape 3.8.2 (Cytoscape Consortium, USA), and Microsoft Excel (Microsoft Office 2021, Microsoft, Redmond, WA). These tools were used to visualize countries, institutions, authors, journals, co-cited articles, keywords, genes, and diseases. Gene and disease information was sourced from the Citexs big data analysis platform (https://www.citexs.com), and relevant visualization maps were constructed to analyze the research status, hotspots, and trends of the study. The gene set was obtained programmatically via the PubTator 3.0 API (https://www.ncbi.nlm.nih.gov/research/pubtator3/api). PubTator 3.0 is an AI-powered biomedical literature resource developed by NCBI, which uses natural language processing to automatically recognize and annotate biomedical entities (including genes and diseases) in the literature. Detailed mathematical definitions of metrics and software parameters are provided in **Supplementary Data**.

## 3 Results

### 3.1 Annual publication trends: a continuously expanding field with sustained growth potential

**Figure 1:**
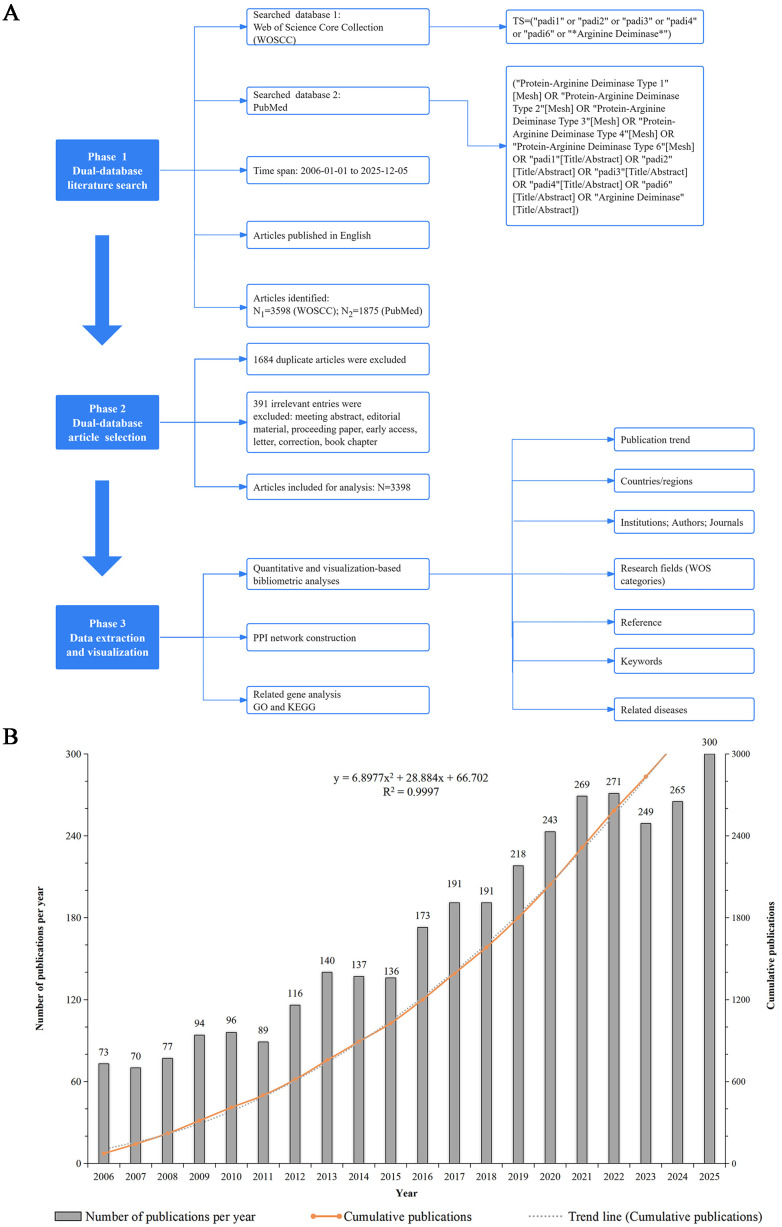
Article selection process and annual publication tendency. **(A)** Article search and selection process; **(B)** Annual publication volume and tendency on PADs from 2006 to 2025.

To understand the overall development of the field, we first analyzed the annual publication trends. As shown in **Figure 1A**, a total of 3398 published papers on the peptidyl arginine deiminase (PAD/PADI) family from January 1, 2006, to December 5, 2025, were included in this study. The average annual number of publications was calculated to be 169.9, showing slight fluctuations with an overall upward trend. The highest growth rate in published papers occurred in 2012 at 30.34%. The peak number of publications was recorded in the partial year of 2025 (up to December 5), with 300 papers, indicating heightened research interest in peptidyl arginine deiminase during that year. A polynomial function was employed to model the annual publication trend: y=6.8977x²+28.884x+66.702 (where R² = 0.9997, x represents the year index with x=0 corresponding to the year 2006, and y represents the predicted cumulative number of publications). While this cumulative approach inherently yields a high coefficient of determination (R² = 0.9997), this is not intended to claim strict predictive accuracy. Rather, the curve serves to demonstrate a steady increase in annual publications, indicating growing research interest in peptidyl arginine deiminase. Consequently, any extrapolations for the coming years should be interpreted cautiously, particularly given the incomplete nature of the 2025 dataset.

### 3.2 Countries/regions and collaborations networks

**Figure 2:**
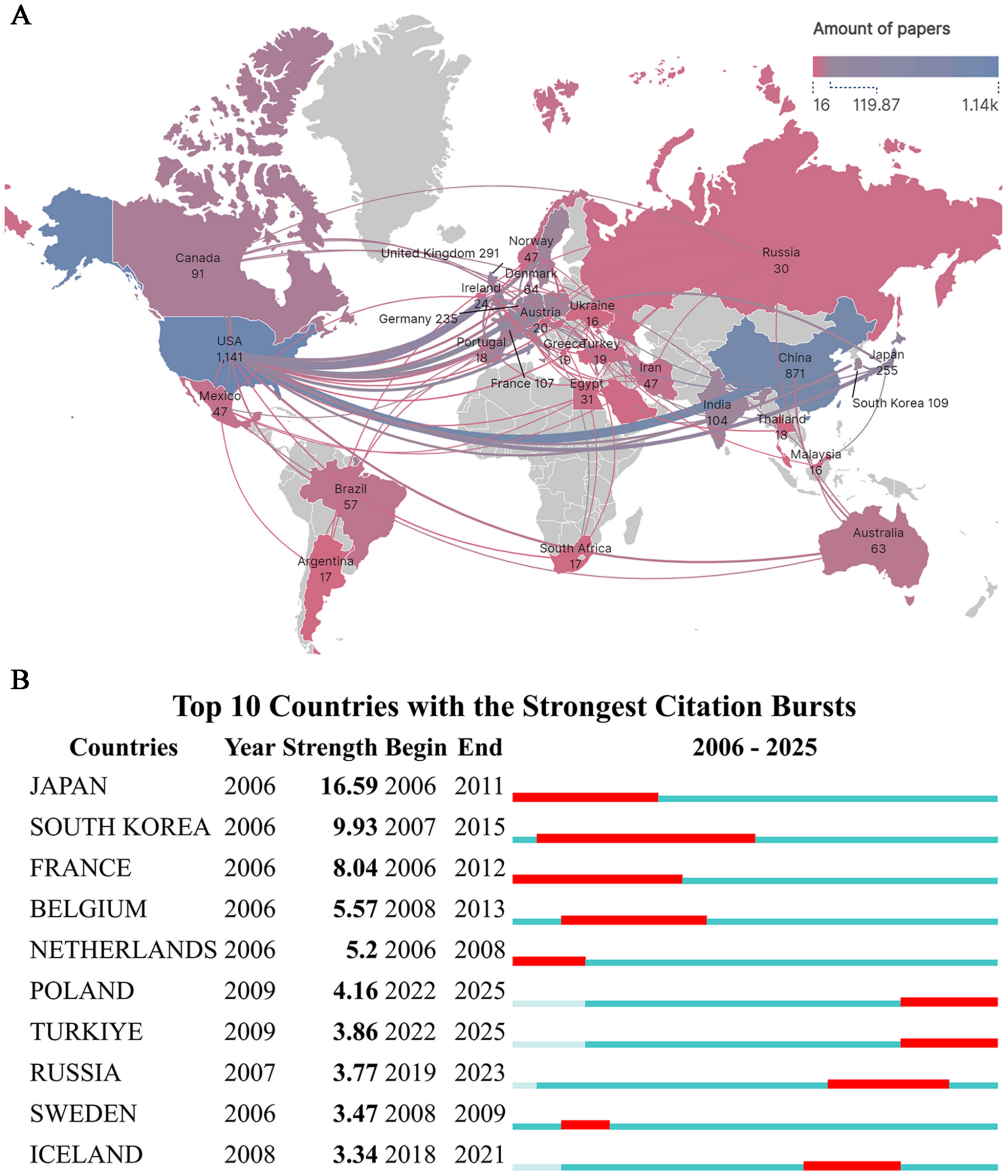
Productivity of research countries/regions and collaborations. **(A)** Co-occurrence and collaboration analysis map of countries/regions. The thickness of connective curves indicates collaboration frequencies between different countries or regions. **(B)** Top 10 countries with the strongest citation burst on PADs from 2006 to 2025. Red lines represent periods of surge.

Analysis at the national level reveals the global distribution of research efforts and collaborative networks. Setting a minimum threshold of 15 publications per country/region, we generated a national collaboration map. In this visualization, gradient colors denote the total number of publications, while line thickness indicates collaboration intensity between countries/regions (**Figure 2A**). **Figure 2A** highlights the United States as the leading contributor, with 1141 published papers, comprising 33.58% of global publications-nearly four times that of the third-ranked country. Following the USA, China and the United Kingdom recorded 871 and 291 articles, respectively. In terms of collaboration intensity, the USA emerges as the most collaborative, followed by the United Kingdom, Germany, and China. Notably, Hungary stands out with the highest average citations per article at 99.62, while Sweden follows closely with 85.68, indicating strong peer recognition. Türkiye and Iran have the most recent average publication dates around 2021, signaling their recent focus on this research area. Using CiteSpace, we identified the top 10 countries experiencing the highest citation outbursts in PAD enzyme family research from January 1, 2006, to December 5, 2025. **Figure 2B** illustrates these countries, highlighting periods of significant citation surges indicated by red areas. Japan experienced the strongest citation burst from 2006 to 2011, peaking at 16.59. South Korea exhibited a significant duration of citation emergence spanning 8 years (2007–2015), underscoring its sustained academic contributions to PAD research. In recent years, citation bursts for Turkiye (2022–2025), Poland (2022–2025), and Russia (2019–2023) showed rapidly growing academic influence.

### 3.3 Research institutions and collaborations analysis

**Figure 3:**
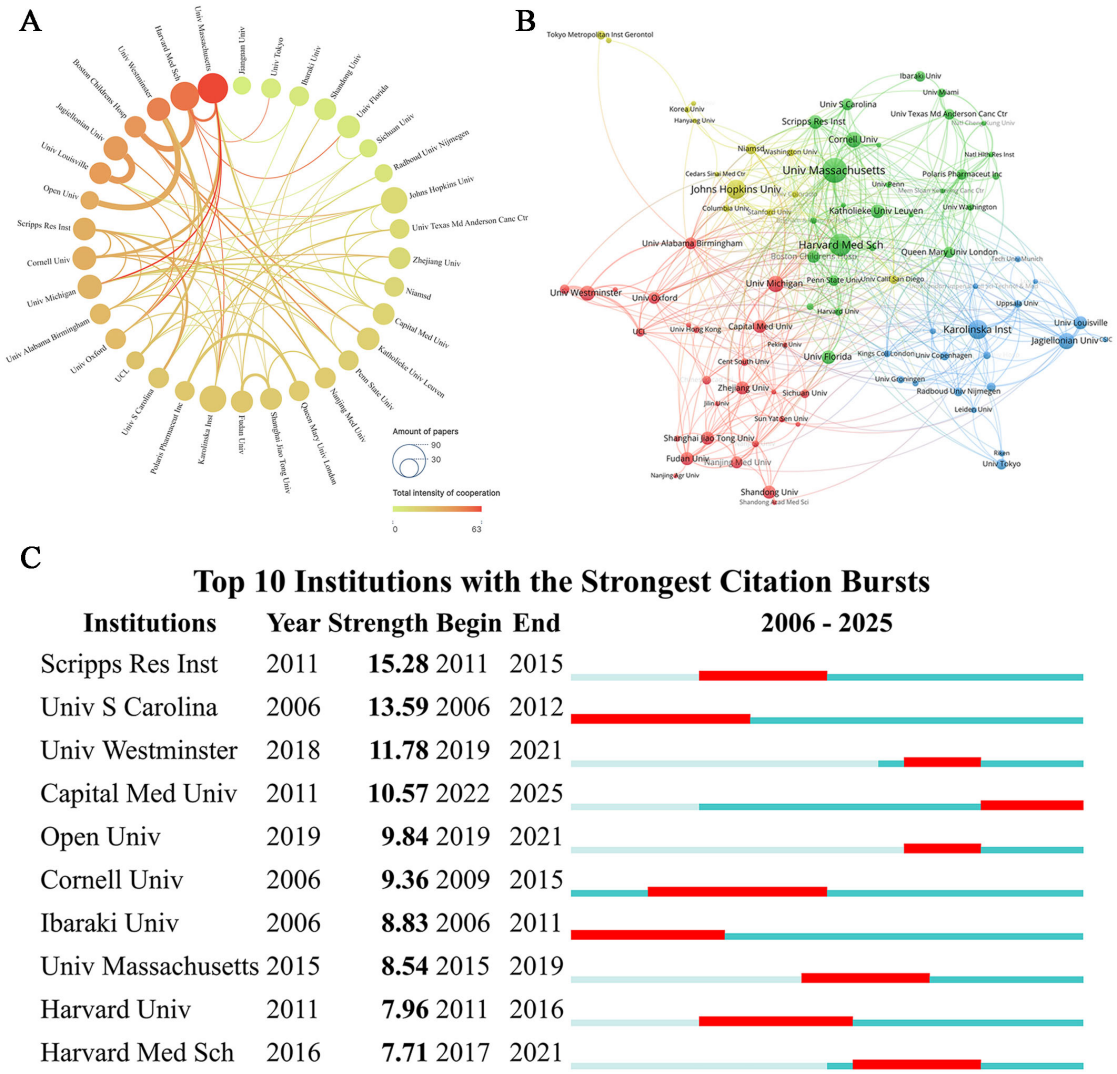
Research institutions and collaborations analysis. **(A)** Chord diagram of cooperation intensity of research institutions. The thickness of connective curves indicates collaboration frequencies between different institutions. **(B)** Clustering and collaboration analysis networks of research institutions. The sizes of the circles represent publication numbers of each institution, and the thickness of the connective curves indicates collaboration frequencies between different institutions. **(C)** Top 10 institutions with the strongest citation burst on PADs from 2006 to 2025. Red lines represent periods of surge.

Using VOSviewer software, we analyzed publishing institutions involved in PAD-related research, identifying a total of 3,200 institutions responsible for 3,398 articles. **Figure 3A** illustrates the cooperation map based on a threshold of 26 publications per institution, while **Figure 3B** presents the clustering map based on a threshold of 15 publications per institution. In **Figure 3A**, each circle represents an institution, with inter-circle lines indicating cooperation, and line thickness denoting cooperation strength. The University of Massachusetts led with the highest number of articles at 73, followed by Harvard Medical School and Karolinska Institute with 66 and 57 publications, respectively. The University of Massachusetts also showed the strongest inclination for collaboration. Notably, Jagiellonian University exhibited the closest collaboration with the University of Louisville. Meanwhile, Pennsylvania State University stood out with the highest average citations per article at 211.84, reflecting significant academic recognition for its publications.

Clustering map of **Figure 3B** based on literature co-citation networks highlighted institutional relationships, classifying institutions into four distinct clusters. The University of Massachusetts, known for its extensive publications and collaborative nature, was classified in the green cluster. Within this cluster, Pennsylvania State University stood out for its highest total citations. Notably, institutions with relatively recent average publication years, such as Capital Medical University, Central South University, Southern Medical University, and the University of São Paulo (averaging around 2022–2023), were grouped into the red cluster, representing emerging forces in this field.

Using CiteSpace, we examined the top 10 institutes with the strongest citation bursts in PAD family research from January 1, 2006, to December 5, 2025 (**Figure 3C**). Scripps Research Institute experienced a peak surge from 2011 to 2015, reaching the highest burst intensity value of 15.28. The University of South Carolina demonstrated the longest duration of citation emergence, spanning from 2006 to 2012—a period of 7 years—indicating sustained academic impact in PAD research over time. Capital Medical University exhibited recent citation emergence (2022–2025), suggesting a concentrated and active research focus on PAD in recent years.

### 3.4 Author productivity and scholar communication

**Figure 4:**
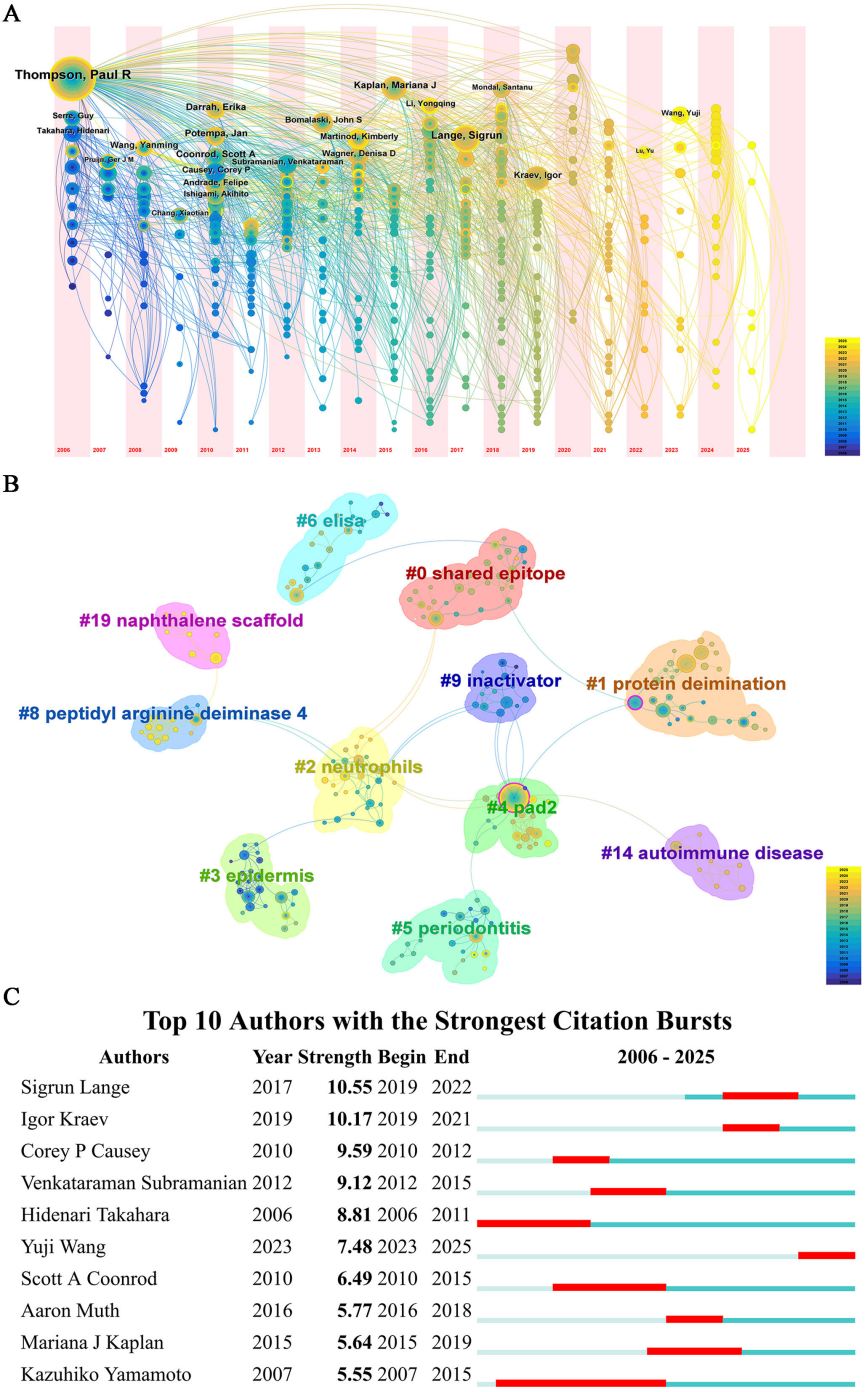
Author and collaboration analysis. **(A)** Timezone view of author publications and cooperative relationships. The position of nodes indicates the year of first publication, and node size corresponds to publication volume. **(B)** Cluster analysis map of authors in PAD research. **(C)** Top 10 authors with the strongest citation bursts on PADs from 2006 to 2025. Red lines represent periods of surge.

We utilized CiteSpace software to visualize the publication timeline and collaborative relationships among authors involved in PAD research from January 1, 2006, to December 5, 2025 (**Figure 4A**). The author timezone view (**Figure 4A**) visualizes the temporal evolution of researchers and their collaborations. Paul R. Thompson is the most prolific author with 122 publications. Authors like Jan Potempa and Erika Darrah, whose activity spans from earlier (purple) to recent (yellow) periods, demonstrate sustained research longevity. The author co-citation clustering network (**Figure 4B**, Modularity Q = 0.9483, Mean Silhouette S = 0.9699) is highly reliable and partitions authors into 11 thematic clusters, including shared epitope, protein deamination, and neutrophils, reflecting specialized research focus within the community. Analysis of authors with the strongest citation bursts (**Figure 4C**) shows that Kazuhiko Yamamoto exhibited the longest duration of citation emergence, spanning 9 years from 2007 to 2015, underscoring sustained academic influence in the field. Sigrun Lange (2019–2022) recently attracted the sharpest surge in attention, while the burst for Yuji Wang (2023–2025) marks them as key recent contributors. This author is affiliated with Capital Medical University, an institution that itself exhibited a prominent citation emergence during an overlapping period (2022–2025). This parallel underscores a concentrated and active research focus on PAD within this team and institution in recent years.

### 3.5 Journal distribution and related field landscape

**Figure 5:**
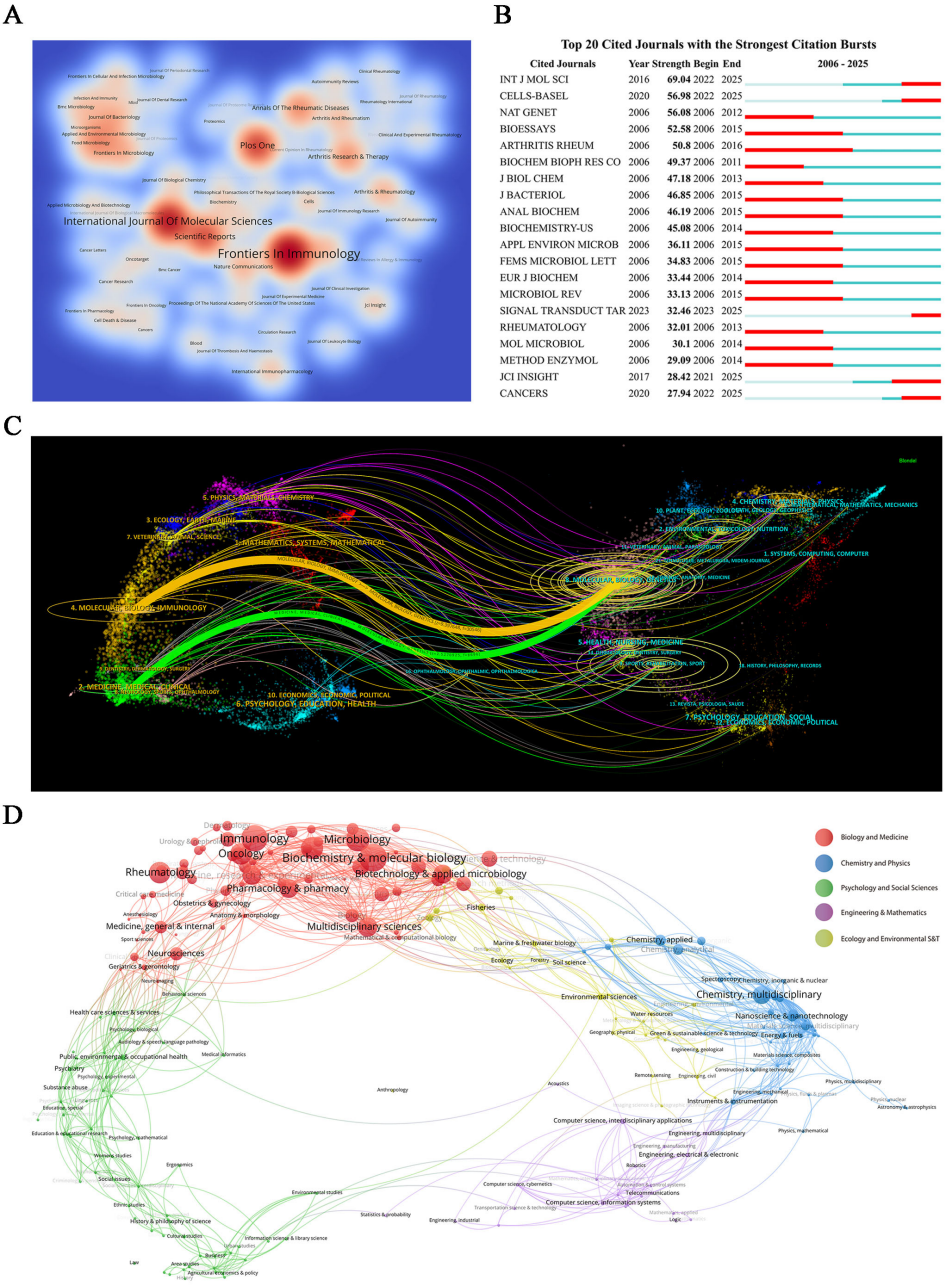
Journal and related field analysis. **(A)** The heatmap of research journals. The color intensity correlates with the publication volume in each journal. **(B)** Top 20 cited journals with the strongest citation burst on PADs from 2006 to 2025. Red lines represent periods of surge. **(C)** Dual-map overlay of journals reflects the interdisciplinary distribution. The figure is divided into two parts: the journals citing others on the left side and the journals cited by others on the right. **(D)** The research field analyses, categorizing articles related to PAD research into 5 major fields.

Journal analysis identifies the primary outlets for research dissemination across 1,144 journals. **Figure 5A** displays a heatmap of publications in different journals, with a minimum threshold of 9 publications per journal. *Frontiers in Immunology* emerged as the journal with the highest publication volume with 119 papers (**Figure 5A**), followed by the *International Journal of Molecular Sciences*, *PLOS ONE*, and *Scientific Reports* with 94, 68, and 60 papers respectively. **Figure 5B** presents the top 20 cited journals with the strongest citation bursts in PAD family research. The *International Journal of Molecular Sciences* exhibited the highest citation burst intensity at 69.04 during the period of 2022–2025, whereas *Arthritis & Rheumatism* showed the longest-lasting citation emergence duration (11 years, 2006–2016), indicating its sustained academic influence in the PAD family research field. Most journals in the top 20 experienced citation surges between 2006 and 2016, with a notable resurgence observed in several journals from 2021 to 2025, suggesting renewed interest and activity in PAD family research during recent years.

**Figure 5C** presents the dual-map overlay analysis chart, offering insights into the interdisciplinary distribution of journals. The chart is divided into sections: journals citing others on the left and those cited by others on the right. PAD family research publications predominantly appear in journals related to Molecular, Biology, Immunology, and Medicine, Medical, Clinical, while these publications are primarily cited in journals focused on Molecular, Biology, and Genetics. Additionally, **Figure 5D** shows the research field analysis, categorizing articles related to PAD research into 5 major clusters. Predominantly, PAD family studies are concentrated in Biology and Medicine, particularly within the sub-field of Biochemistry & Molecular Biology, accounting for the highest frequency (535). Immunology (465), Microbiology (320), Cell Biology (271), and Oncology (254) also feature prominently in the analysis (**Figure 5D**). The analysis of journals and related fields offers insights into publication patterns, emerging trends, interdisciplinary connections, and future research directions.

### 3.6 Co-citation references and burst analysis

**Figure 6:**
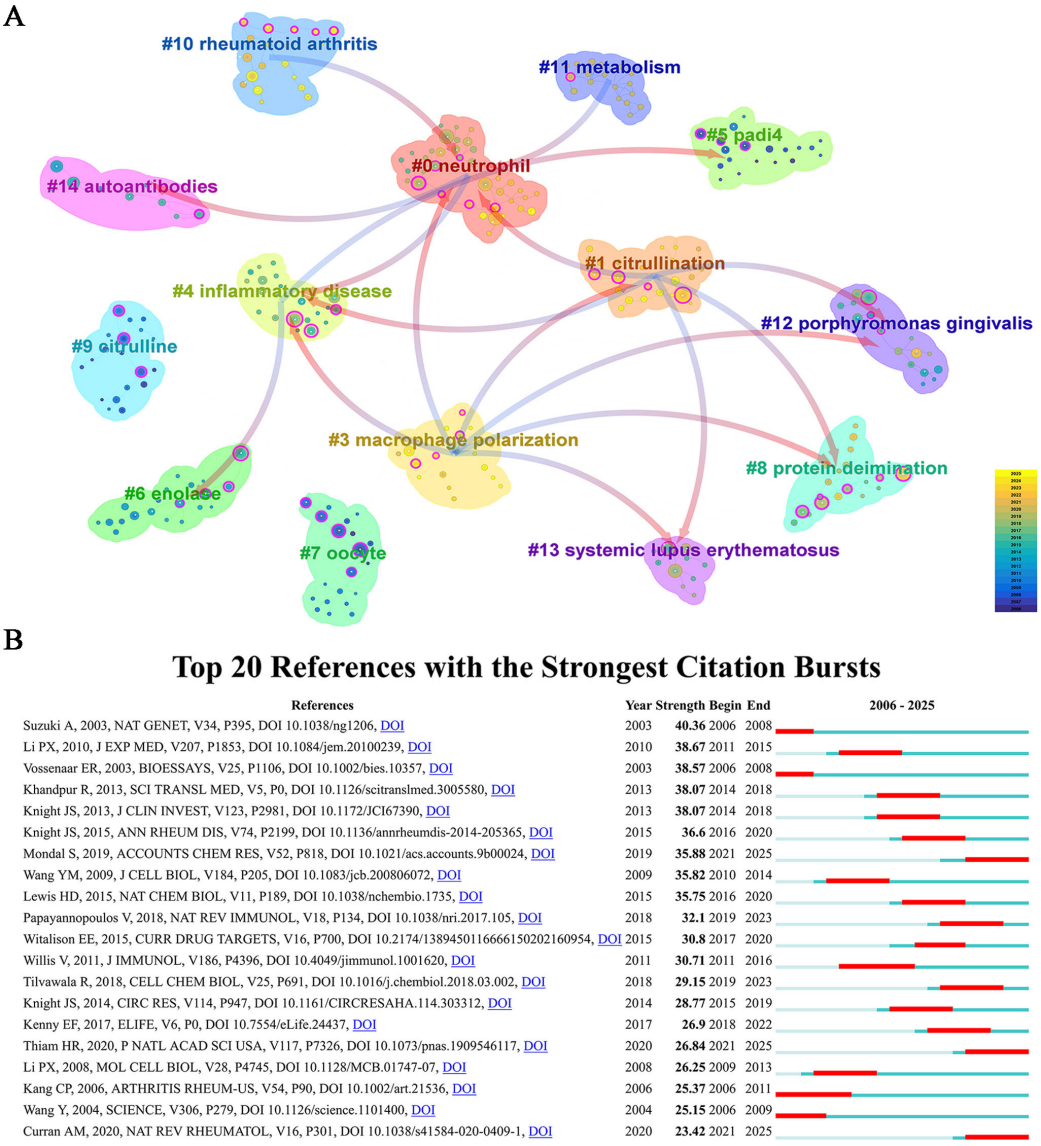
Co-citation reference and burst analysis. **(A)** The co-citation analysis network of references, clustered by research topics. **(B)** Top 20 references with the strongest citation bursts on PADs from 2006 to 2025. Red lines represent periods of citation surge.

The publication co-cited clustering network (**Figure 6A**) analyzes derivative and citation relationships across different clusters. CiteSpace calculates Modularity (Q) and Mean Silhouette (S) values to assess clustering significance and structural clarity. Typically, Q values above 0.3 and S values above 0.5 indicate significant and reasonable clustering, respectively. In this analysis (**Figure 6A**), the cluster modularity value Q is 0.8844, and the mean silhouette value S is 0.9812, comfortably meeting these criteria, thus confirming the clustering’s high significance and credibility. **Figure 6A** illustrates that co-cited documents are categorized into 17 distinct clusters. The major clusters include neutrophil, citrullination, macrophage polarization, inflammatory disease, padi4, enolase, oocyte, protein deimination, citrulline, rheumatoid arthritis, metabolism, Porphyromonas gingivalis, systemic lupus erythematosus, and autoantibodies. These categories highlight a diverse range of topics, from cellular mechanisms to specific autoimmune diseases and pathogens. Inter-cluster links suggest foundational relationships where earlier research topics provide the basis for emerging fields. Analysis of references with strong citation bursts (**Figure 6B**) tracks the shift of knowledge hotspots: the seminal paper by Suzuki A et al. (2003) on PADI4 and rheumatoid arthritis had the strongest early burst (intensity 40.36, 2006–2008). Notably, over half of the top-cited burst references experienced their surge after 2014, and recent high-impact works like Mondal S (2019) (*Accounts Chem Res*), Thiam HR (2020) (*PNAS)*, and Curran AM (2020) (*Nat Rev Rheumatol*) continue to emerge (2021–2025), indicating sustained innovation in areas like novel inhibitors and the role of PADs in neutrophil biology.

### 3.7 Keyword analysis and research frontiers

**Figure 7:**
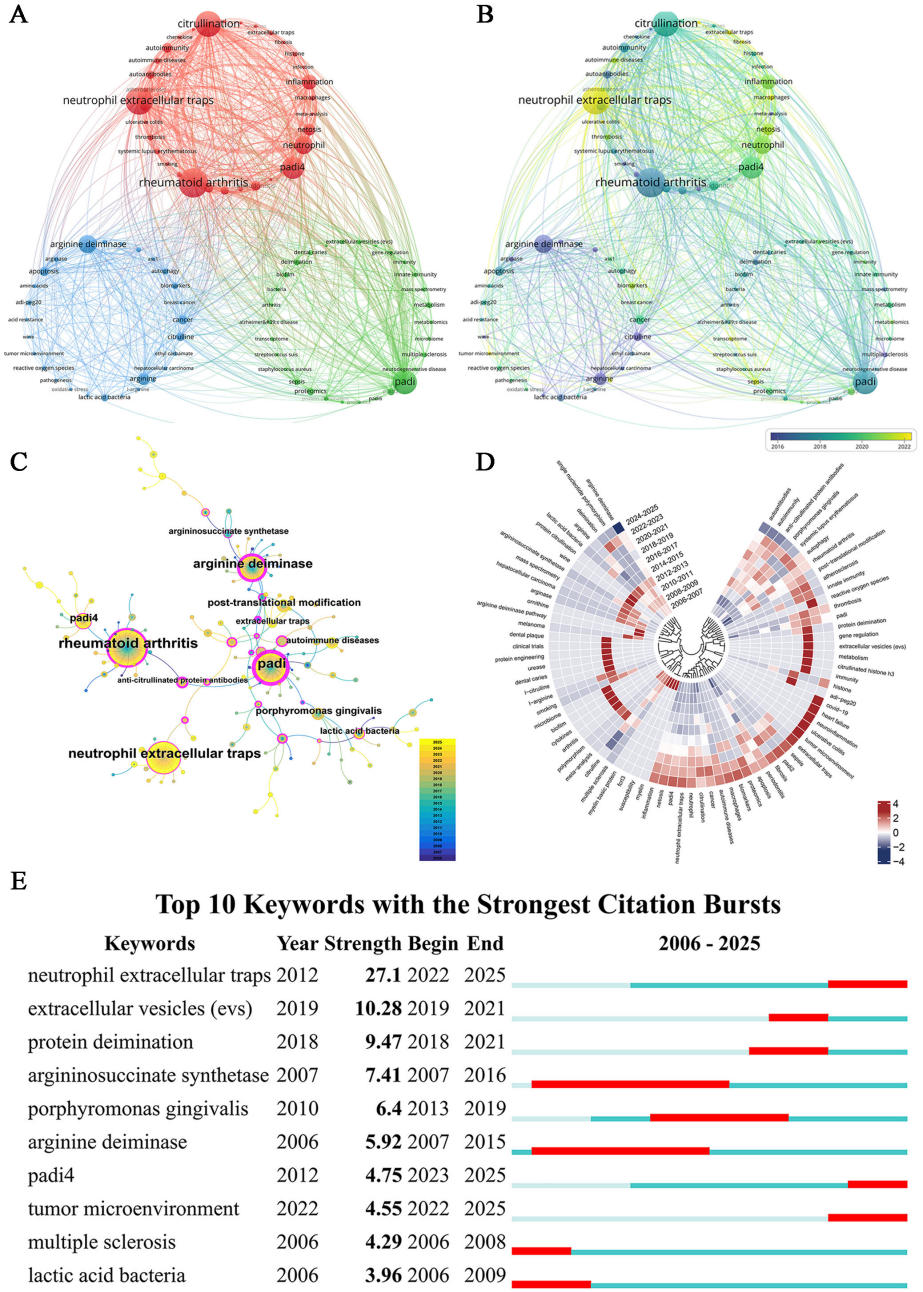
Keyword analysis. **(A)** Co-occurrence clustering graph of keywords. The three colors represent different research directions. **(B)** The time-frequency map of keywords showing the evolution of research topics from early to recent years. **(C)** The frequency co-occurrence analysis network. **(D)** The heatmap analysis of PAD family-related keywords from 2006 to 2025. **(E)** The top 10 keywords with the strongest citation bursts on PADs from 2006 to 2025. Red lines represent periods of surge.

Keyword analysis dynamically captures the shift of research hotspots. Out of 5,845 identified keywords, 91 met the minimum occurrence threshold of 13 and were selected for visualization (**Figure 7A**). Co-occurrence clustering (**Figure 7A**) identifies three major thematic groups: Immunology & Autoimmune Diseases (red), Enzyme Family & Biological Processes (green), and Arginine Metabolism & Oncology (blue). **Figure 7B** displays the time-frequency map of keywords, where the color scale indicates the average publication year. Keywords such as arginine and arginine deiminase appeared relatively early (around 2016), laying the biochemical foundation of the field. In contrast, terms like neutrophil extracellular traps, fibrosis, tumor microenvironment, and COVID-19 have emerged prominently after 2021, highlighting the most recent research trends and hotspots. **Figure 7C** illustrates the frequency co-occurrence analysis using CiteSpace. Rheumatoid arthritis remains the most frequently cited keyword (384), followed by neutrophil extracellular traps (280) and PADI (271), underscoring the central role of these topics in the network. **Figure 7D** presents a heatmap analysis of PAD family-related keywords from January 1, 2006, to December 5, 2025. It shows that traditional topics like rheumatoid arthritis have maintained high popularity historically, while emerging topics such as COVID-19, PADI2, and extracellular traps have shown increasing heat in recent years. **Figure 7E** lists the top 10 keywords with the strongest citation bursts. Notably, “neutrophil extracellular traps” exhibited the highest citation burst intensity at 27.1, with a significant surge from 2022 to 2025. “Argininosuccinate synthetase” showed the longest citation emergence period, spanning 10 years from 2007 to 2016. Furthermore, “PADI4” and “tumor microenvironment” have shown strong citation bursts in the most recent three years (2023–2025 and 2022–2025, respectively), suggesting that the specific isoforms of PADs and their roles in the cancer microenvironment are becoming key focuses for future research. However, it is important to note that these citation bursts reflect rapid surges in academic attention and research momentum, which do not automatically equate to proven biological importance or clinical relevance.

### 3.8 Gene and pathway enrichment analysis and disease associations

**Figure 8:**
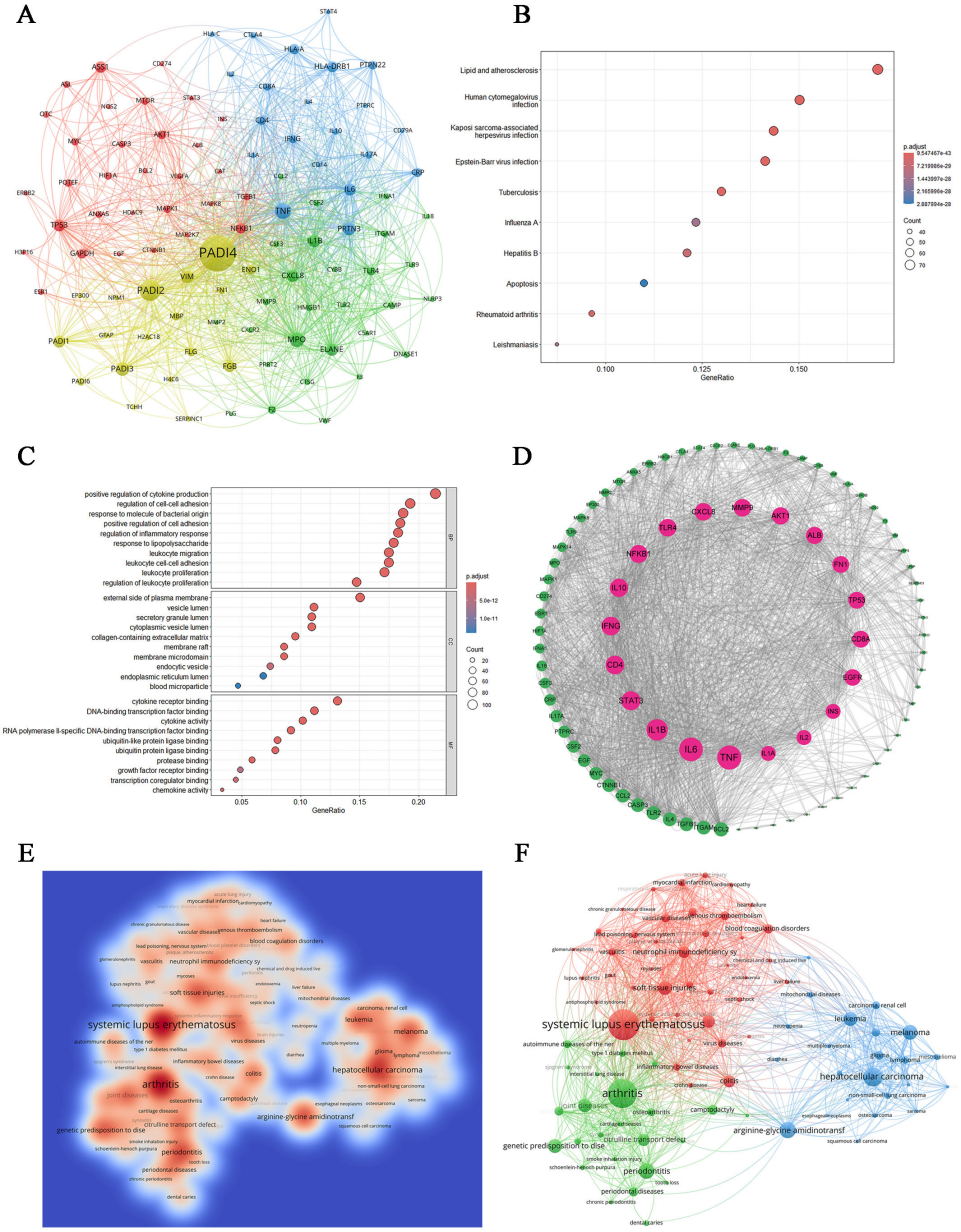
Gene and pathway enrichment analysis. **(A)** Co-occurrence clustering graph of genes related to the PADs. **(B)** KEGG pathway enrichment analysis bubble chart. **(C)** GO enrichment analysis bubble chart. **(D)** The core protein PPI network arranged by degree. **(E)** The heatmap of related diseases. **(F)** Cluster analysis network of related diseases. These enrichment signals reflect co-occurrence patterns in the published literature and do not imply direct biological causation.

Using VOSviewer software on the CiteXS data platform, we analyzed 4,560 genes extracted from 3,398 articles related to the PADI gene family. **Figure 8A** illustrates the visual graph of cluster analysis, depicting various gene clusters categorized by different colors, with a minimum occurrence threshold of 37. Each node represents a gene, with the size of the node indicating the frequency of gene occurrence, and the thickness of connecting lines reflecting the strength of relationships between genes. The highest popularity gene, PADI4, is classified in the yellow cluster. PADI4 encodes an enzyme that catalyzes the conversion of arginine to citrulline residues, influencing protein conformation and function. It plays a significant role in inflammation regulation, particularly in the formation of neutrophil extracellular traps (NETs) and the regulation of NETosis, as well as in various tumor-related diseases. PADI2, the second most popular gene, is also located in the yellow cluster (closely frequently co-occurring in the literature with PADI4). Like PADI4, PADI2 is a member of the PADI gene family but exhibits different substrate specificity and tissue-specific expression patterns; it is primarily frequently co-occurs in the literature with autoimmune diseases, inflammatory disorders, and the progression of neurodegenerative diseases. In the red cluster, TP53 emerges as the gene with the highest research enthusiasm. TP53 plays a crucial role in cell cycle regulation, apoptosis, senescence, DNA repair, and metabolic changes. In the green cluster, MPO (Myeloperoxidase) stands out as a central gene, involved in pathways related to the innate immune system and often studied alongside PADI4 in the context of neutrophil function. The blue cluster is centered on TNF and IL6, highlighting the critical role of cytokines in the inflammatory response regulated by PADI.

KEGG pathway enrichment analysis (illustrated in **Figure 8B**) revealed that research on the PADI gene family members is significantly associated with several critical signaling pathways. The X-axis represents the GeneRatio, where a higher value indicates a greater association of genes with the pathway. Significant pathways include the Lipid and atherosclerosis pathway, and the Human cytomegalovirus infection pathway. Importantly, because our input gene set is inherently skewed by highly studied inflammatory hub genes (e.g., TP53, IL6, TNF, MPO), these KEGG enrichments primarily reflect literature co-occurrence signals rather than verified, direct functional associations with PAD biology. They essentially indicate that current PAD research frequently overlaps with broader immunological and metabolic studies in the literature, driven by the publication popularity of these hub genes.

GO enrichment analysis of the related genes is shown in **Figure 8C**. Enrichment analysis revealed significant associations in three categories: In the Biological Process (BP) category, genes primarily show literature enrichment signals for positive regulation of cytokine production, regulation of cell-cell adhesion, and response to molecules of bacterial origin. For Cellular Components (CC), the most significant terms include the external side of the plasma membrane, vesicle lumen, and secretory granule lumen. In Molecular Functions (MF), genes show strong literature enrichment signals for cytokine receptor binding, DNA-binding transcription factor binding, and cytokine activity. These findings indicate key biological functions and interactions of the analyzed genes in the context of PAD/PADI family research.

Using the Citexs big data platform, we imp orted the top 100 proteins frequently appearing in articles related to PAD/PADI research into the STRING platform to extract protein-protein interaction (PPI) network information. **Figure 8D** illustrates the core protein PPI network, arranged in ascending order according to their degree values. The top 10 proteins identified in this network are TNF, IL6, IL1B, STAT3, CD4, IFNG, IL10, NFKB1, TLR4, and CXCL8. These proteins are likely pivotal in understanding the molecular mechanisms underlying inflammation and related diseases within the context of PAD research. Furthermore, we extracted a total of 2,183 diseases mentioned in the literature. Diseases with at least 40 occurrences were visualized using VOSviewer software (**Figure 8E**). The heatmap analysis reveals that systemic lupus erythematosus (SLE), arthritis, and hepatocellular carcinoma are the most frequently co-mentioned diseases in PAD-related literature. **Figure 8F** shows the clustering network of these diseases. In the red cluster, systemic lupus erythematosus stands out with high research frequency, grouped with autoimmune and vascular conditions like venous thromboembolism. In the blue cluster, arthritis emerges as the central node, including related conditions such as periodontitis and joint diseases, all frequently co-occurring in the literature with chronic inflammation. The green cluster highlights research enthusiasm for malignancies, particularly hepatocellular carcinoma, leukemia, and melanoma. It should be emphasized that these results reflect areas of concentrated academic attention and research hotspots identified via bibliometric text mining, rather than direct biological causation. The highly studied hub genes (e.g., TP53, TNF, IL6) may appear prominent due to their general research popularity rather than specific PAD-related functions. Our analysis does not adjust for this publication bias.

Interestingly, many diseases show significant correlations with the PAD family despite not having been extensively studied in conjunction before. This suggests a potentially fruitful area for future research, emphasizing the high research value and interdisciplinary connections of PAD-related diseases.

## 4 Discussion

Posttranslational modifications (PTMs) are important epigenetic mechanisms that regulate protein function by altering their molecular structures (2, 14, 15). They play key roles in various physiological processes and in the pathophysiology of diseases, particularly inflammation and cancer (2, 16–18). Citrullination is a specific type of PTM catalyzed by the peptidyl arginine deiminase (PAD) enzyme family, which consists mainly of PAD1–4 and PAD6, each exhibiting tissue-specific expression (19, 20). The activity of PAD enzymes depends on increased calcium concentrations (21). Compared to other types of PTMs, research on citrullination is relatively limited. Therefore, identifying the most relevant data is crucial for understanding the current state of research and identifying potential future directions. In this study, we conducted a retrospective analysis of PADs over the last 20 years using the Web of Science Core Collection (WoSCC) and PubMed to ensure maximum data coverage. The initial search yielded 3,598 articles from WoSCC and 1,875 from PubMed, resulting in a final corpus of 3,398 publications. Over the past two decades, the publication rate has consistently increased, peaking in the partial year of 2025 (up to December 5), with the highest growth rate observed in 2012. The United States and China have contributed the greatest number of studies on PADs, while Hungary has the highest average citation count per article. These findings reveal a growing interest in PAD research, with the United States maintaining a leading position, although Hungary’s contributions are highly recognized among peers. At the institutional level, the University of Massachusetts leads in both the number of publications and collaborative efforts. The number of publications reflects the level of interest and enthusiasm for PAD research, while citation counts indicate academic influence in the field. Citation prominence, defined as a significant increase in the number of citations over a specific period, shows that Japan and South Korea have maintained prominence for the longest duration. Recently, research on PADs in Turkiye, Poland, and Russia has gained increased attention, reflecting a growing academic influence due to their high citation prominence. Pennsylvania State University has the highest average citation count per article and total citation count, indicating a high level of academic recognition for its published work. Notably, Capital Medical University has experienced a surge in citations, and it is also the institution with the most recent average publication time, underscoring its strong focus on PAD research in recent years. In the realm of PAD research, the most prolific author is Paul R. Thompson from the University of Massachusetts, aligning with the institution’s leadership in both publications and citations (22–24). Specifically, the author team of Yuji Wang and Yu Lu from Capital Medical University has been identified as key recent contributors, demonstrating a significant citation burst from 2023 to 2025. Their impactful work is highly representative of a shifting research frontier, being concentrated on the development of novel PAD inhibitors, as evidenced by studies on enhancing the metabolic stability and potency of PAD4 inhibitors (25, 26) and reviewing their role in cancer therapy (27). This thematic focus aligns with the observed citation emergence of their institution, Capital Medical University (2022–2025), and underscores a broader trend in the field toward intensifying research on PADs as promising drug targets. Overall, these data suggest a continuous increase in enthusiasm for PAD research, accompanied by enhanced collaboration and communication among countries, institutions, and authors. This indicates a vibrant and open research atmosphere in this field.

Our analysis covers a total of 3,398 articles related to PADs, published in 1,144 journals. The journal with the highest number of published articles is Frontiers in Immunology, which primarily focuses on fields such as molecular biology and immunology. Citation trend analysis further identified rheumatoid arthritis (RA) as the most prominent research focus, which is highly relevant to the core function of PADs: citrullination. Interestingly, RA is a classic autoimmune disorder that affects multiple synovial tissues and joints throughout the body, leading to the upregulation of PAD2 and PAD4 expression in various organs and tissues. This upregulation induces increased citrullination of key proteins, such as ACPA and histone H4, and these circulating citrullinated proteins not only exacerbate RA-related myocardial and renal damage but also act as autoantigens to trigger systemic inflammation. This line of investigation underscores the dual role of PAD-mediated citrullination in pathological progression, laying the foundation for subsequent explorations of PADs in broader disease contexts (28–30). The COVID-19 pandemic has further amplified interest in PADs, with keywords such as “COVID-19” and “immunology” receiving significant attention in recent research (31).

In recent years, research has gradually expanded beyond classic autoimmune models to more diverse inflammatory scenarios, driven by the systemic distribution and mobility of immune cells. A striking rise in citations of keywords related to neutrophil extracellular traps (NETs) highlights this trend. Notably, “neutrophil extracellular traps” exhibited the highest citation burst intensity (27.1) in our keyword analysis. As immune cell-mediated forms of cell death, NETs play a crucial role in bacterial clearance during inflammation, but also participate in disease progression, with PADs (especially PAD2 and PAD4) identified as critical regulators (32–34). This link between PADs and trap-mediated inflammation has further led to recent studies increasingly linking PADs to sepsis. Mechanistically, PAD2 and PAD4 directly contribute to sepsis progression by regulating NET formation and immune signaling. NETs, extracellular fiber networks composed of citrullinated histones, DNA, and proteases, play a dual role in sepsis: while they initially help trap and eliminate pathogens, excessive NET release (driven by PAD4-mediated histone citrullination) exacerbates endothelial barrier dysfunction, intravascular thrombosis, and disseminated intravascular coagulation (DIC). Beyond this direct regulation of NETs and immune signaling, PADs may also intersect with key signaling pathways implicated in sepsis pathogenesis, such as the NLRP3 inflammasome and STING pathway (35, 36). And our reference co-citation analysis identified “#0 neutrophil” as the largest cluster, further supporting the strong correlation between PADs and inflammatory diseases, highlighting their potential for future research.

These trends indicate that PAD research continues to evolve and remains highly relevant in contemporary scientific discourse. Among the PADI gene family, PADI4 is the most extensively studied, followed by PADI2, both clustering in the yellow group associated with enzymatic functions. Co-occurrence clustering analysis of related genes revealed that TP53 (red cluster) and MPO (green cluster) are also central nodes, suggesting potential molecular mechanisms linking inflammation and tumorigenesis. Most molecules associated with PADI4 are broadly linked to inflammation and tumors. Our enrichment analysis yielded strong literature co-occurrence signals in domains such as lipid and atherosclerosis, viral infections (e.g., Cytomegalovirus), and inflammatory responses (37, 38). However, it is crucial to note that these signals are heavily driven by the presence of pan-inflammatory hub genes in the text-mining dataset, thus reflecting interdisciplinary research trends and publication bias rather than specific PAD-mediated biological mechanisms. In recent years, PADI4 and the tumor microenvironment have shown strong citation bursts, indicating a shift towards oncology (39, 40). Notably, PADI2 has also garnered significant attention (41); given its broader tissue expression, it may involve distinct molecules linked to conditions such as arrhythmia and venous thromboembolism, which appeared in the disease clustering analysis. This highlights the unique molecular roles of different PAD isozymes in diverse pathologies.

Recognizing these distinct pathogenic roles has directly propelled advancements in pharmacological targeted therapies. To frame our bibliometric trends within this pharmacological context, it is crucial to highlight the evolution of PAD inhibitors, which remain central to clinical translation. Early foundational research heavily relied on pan-PAD inhibitors, such as Cl-amidine and BB-Cl-amidine, which were instrumental in establishing the pathogenic role of citrullination in autoimmune and inflammatory models (42). However, to minimize systemic off-target effects and elucidate the specific mechanisms of individual isozymes mentioned above, the field has rapidly shifted toward the development of isoform-specific inhibitors. For example, the emergence of highly selective PAD4 inhibitors (such as GSK199) has provided a critical pharmacological tool that may have driven the recent surge in research into the specific roles of PAD4 in NETs and the tumor microenvironment—both of which emerged as the research areas with the most significant increase in citations in our analysis (43). Similarly, the emergence of PAD2-specific inhibitors (such as AFM-30a) may also have facilitated targeted research into PAD2-driven transcriptomic regulation and related conditions (44). This ongoing pharmacological transition from broad-spectrum to targeted inhibition drives the shifting research frontiers. Specifically, clinical trials involving pan-inhibitors or specific inhibitors targeting these different subtypes of the PAD family need to be designed and conducted in the future in order to provide patients with treatments that offer genuine clinical value.

This study’s visualizations, generated through bibliometric analysis of a dual-database dataset, provide valuable insights for practitioners in the field, including information on prominent research institutions, leading scientists, influential journals, and trending topics. By highlighting research popularity and identifying active countries, institutions, and researchers in the field of PADs, this study serves as a guide for collaboration and knowledge sharing among interested researchers. Importantly, the bibliometric analysis allows us to uncover unexplored yet promising areas, such as the emerging role of PADs in metabolic diseases and viral pathogenesis.

However, it is essential to interpret these bibliometric trends with caution. While citation bursts identify hot-spots like NETs and the tumor microenvironment, they reflect academic focus rather than a resolution of the underlying biological complexity. Future research must bridge these quantitative trends with deep mechanistic studies to overcome existing scientific challenges.

## 5 Limitations

This study has several limitations that should be acknowledged. Firstly, although we employed a dual-database strategy merging Web of Science and PubMed, to maximize coverage, it does not cover all journals worldwide; non-English publications, non-indexed journals, regional outlets, and conference proceedings may be under-represented. Secondly, recent high-quality studies, particularly those published in 2024 and 2025 regarding emerging targeted therapies or immunotherapies, may not yet have accumulated sufficient citations to reflect their true academic impact. Thirdly, a sensitivity check revealed that our PubMed search string missed 431 papers (44%) specifically using the term “peptidylarginine deiminase”. Although our dual-database strategy likely rescued many of these via Web of Science, we did not quantify this exact overlap, leaving a potential gap in our final corpus. Fourthly, we employed a relatively broad search strategy in the WoS and PubMed databases, which resulted in an estimated 89 papers (2.8%) of residual contamination from unrelated literature in WoS; we retained them in the final bibliometric analysis to prevent subjective exclusion bias and this figure serves strictly as an estimate of the background noise within our dataset. Finally, the absence of statistical data on PADs research may also introduce biases in the analysis. Despite these limitations, this study provides valuable reference points for understanding global research hotspots and trends in PADs.

## 6 Conclusion

The research maps the evolving landscape of PAD research over the past two decades. Our findings highlight a rapid expansion of literature, particularly in inflammatory and tumor-related diseases. However, cooperation between institutions and countries remains limited, although trends indicate increasing cooperation. Overall, this study identifies major research hotspots and evolving trends within the PAD field, helping to guide future research directions and providing an objective, data-driven reference for tracking new frontiers in the field, rather than drawing biological conclusions.

## Data Availability

The original contributions presented in the study are included in the article/**Supplementary Material**. Further inquiries can be directed to the corresponding authors.
