# Supplementary material for: Bibliometric and visualization analysis of arginine deiminase research from 2006 to 2025: trends, collaboration networks and emerging frontiers
Source: Front Immunol. 2026 Jul 22;17:1784910. doi: 10.3389/fimmu.2026.1784910 (PMC13437694; doi:10.3389/fimmu.2026.1784910)
Supplement: Supplementary file 1 [file SupplementaryFile1.zip › Supplementary Data/Method and Definition.docx]

**Method**

Figure 2 A

Counting method: Full counting method (the default setting for co-authorship analysis in VOSviewer, which counts the full weight of each publication for all participating countries).

Normalization method: Association strength normalization (the default normalization method in VOSviewer, which standardizes link strengths to eliminate the bias caused by differences in node size).

Clustering: No clustering was performed for this visualization, as this figure focuses on displaying the overall geographical distribution and collaboration intensity of high-output countries.

Threshold: To ensure the readability of the figure, we set a minimum publication threshold of 15 documents per country. Among the total 96 countries that contributed to the 3398 included publications, 21 countries met this threshold and were included in the visualization.

Figure 2 B

Burst detection algorithm: Kleinberg’s burst detection algorithm, the standard algorithm implemented in CiteSpace to identify sudden surges in citation frequency.

Burst detection parameters: We used the default parameter settings for burst detection, with the tuning coefficient γ (gamma) set to 0.5 and the minimum burst duration set to 1 year, which balances the sensitivity and specificity of burst detection.

Time range: The analysis covered the entire time span of our study: January 1, 2006 to December 5, 2025.

Threshold: We ranked all included countries by their burst strength, and selected the top 10 countries with the strongest citation bursts for visualization.

Figure 3 A

Counting method: Full counting method.

Normalization method: Association strength normalization.

Clustering: No clustering was performed for this figure, which focuses on displaying the collaboration intensity between institutions with the highest publication output.

Threshold: We set a minimum publication threshold of 26 documents per institution to filter the most productive institutions. Among the total 3200 institutions that contributed to the included publications, 28 institutions met this threshold and were included in the visualization.

Figure 3 B

Counting method: Full counting method.

Normalization method: Association strength normalization.

Clustering algorithm: The clustering was performed using the VOS clustering algorithm (the default clustering algorithm implemented in VOSviewer), a non-overlapping clustering method that groups nodes with close co-occurrence relationships into clusters by minimizing the weighted sum of distances between nodes, to identify the research community structure.

Threshold: We set a minimum publication threshold of 15 documents per institution to include more institutions for community structure analysis. A total of 76 institutions met this threshold and were included in the clustering analysis and visualization.

Figure 3 C

Burst detection algorithm: Kleinberg’s burst detection algorithm.

Burst detection parameters: Default parameters with γ = 0.5 and minimum burst duration = 1 year.

Time range: January 1, 2006 to December 5, 2025.

Threshold: We ranked all institutions by their burst strength and selected the top 10 institutions with the strongest citation bursts for visualization.

Figure 4 A

Counting method: Full counting method.

Time slicing: We divided the entire time span (2006-2025) into 1-year time slices, the standard setting for timezone visualization to display the temporal evolution of author contributions.

Selection criterion & network parameters: The g-index algorithm was adopted for node selection with the key parameter set as k=25. Other customized CiteSpace parameters were configured as follows: LRF=3.0, L/N=10, LBY=5, e=1.0.

Figure 4 B

Clustering algorithm: The clustering was performed using the log-likelihood ratio (LLR) algorithm, the default clustering method in CiteSpace, which extracts cluster labels and identifies cluster structures with high semantic clarity.

Time slicing: The entire time span (2006-2025) was divided into 1-year time slices.

Selection criterion & network construction parameters: The g-index algorithm was adopted for node selection with the key parameter set as k=17. Other customized CiteSpace parameters for network construction were configured as follows: LRF=3.0, L/N=10, LBY=5, e=1.0.

Cluster validity metrics: To evaluate the reliability of the clustering results, we calculated the modularity Q value and the mean silhouette S value.

Figure 4 C

Burst detection algorithm: Kleinberg’s burst detection algorithm.

Burst detection parameters: Default parameter configuration was adopted, with the tuning coefficient γ = 0.5 and a minimum burst duration of 1 year, ensuring balanced detection sensitivity and accuracy.

Time range: January 1, 2006 to December 5, 2025, consistent with the unified time span of all bibliometric analyses in this study.

Threshold: All authors were ranked according to their citation burst strength, and the top 10 authors with the most prominent citation burst trends were selected for visualization.

Figure 5 A

Counting method: Full counting method, which fully counts the publication contribution of each eligible journal to ensure accurate statistical quantification of journal output.

Normalization method: Association strength normalization, which standardizes node correlation data and eliminates statistical bias caused by differences in journal publication volume.

Clustering: No clustering was performed for this heat map visualization, which focuses on intuitively presenting the publication distribution and output intensity of core journals in the research field.

Threshold: A minimum publication threshold of 9 documents per journal was set to filter core productive journals and control the number of visualized nodes. A total of 1144 journals contributed 3398 publications, and only journals meeting the minimum publication criterion were retained and displayed in the heat map to guarantee visualization clarity.

Figure 5 B

Burst detection algorithm: Kleinberg’s burst detection algorithm.

Burst detection parameters: Default parameter settings were applied, with the tuning coefficient γ (gamma) set to 0.5 and the minimum burst duration set to 1 year to balance detection sensitivity and specificity.

Time range: January 1, 2006 to December 5, 2025, consistent with the unified analysis time span of all bibliometric analyses in this study.

Threshold: All journals involved in the analysis were ranked by their citation burst strength, and the top 20 journals with the most prominent citation burst trends were selected for visualization.

Figure 5 C

Time range: January 1, 2006 to December 5, 2025, consistent with the unified analysis time span of all bibliometric analyses in this study.

Counting & mapping rules: The dual-map overlay consists of two panels: the left panel displays citing journals (source disciplines of published papers), and the right panel displays cited journals (knowledge base disciplines that the research relies on). Each node in the graph represents an individual academic journal. The curved connecting lines between the left and right panels represent cross-journal citation links, which intuitively reveal the interdisciplinary knowledge flow and citation inheritance relationships of the research field.

Core disciplinary distribution parameters: The quantitative indicators z-value (centrality coefficient) and f-value (citation frequency) were adopted to identify dominant disciplinary clusters.

Figure 5 D

Data source: All bibliographic records were retrieved from the Web of Science Core Collection database.

Counting method: Full counting method, which equally weights each publication to ensure unbiased statistical counting of research field distribution.

Normalization method: Association strength normalization, applied to standardize the co-occurrence relationships of research fields and eliminate statistical deviations caused by unbalanced field output.

Clustering algorithm: VOS clustering algorithm (default algorithm of VOSviewer), which groups research fields with close semantic and disciplinary correlation into independent clusters by optimizing network distance minimization.

Clustering result: The overall research literature was automatically classified and visualized into 5 distinct major research field clusters by the algorithm, reflecting the main disciplinary distribution and research framework of this domain.

Threshold setting: No additional artificial publication threshold was set for field clustering. All valid literature discipline categories derived from the Web of Science Core Collection were included to comprehensively present the overall field distribution characteristics of the research.

Figure 6 A

Time range: January 1, 2006 to December 5, 2025, consistent with the unified analysis time span of all bibliometric analyses in this study.

Time slicing: The full time span was divided into consecutive 1-year time slices for consistent network construction and clustering analysis.

Selection criterion & network construction parameters: The g-index algorithm was adopted for node selection with the key parameter set as k=3. Other customized CiteSpace parameters were configured as follows: LRF=3.0, L/N=10, LBY=5, e=1.0.

Clustering algorithm: Log-likelihood ratio (LLR) algorithm

Cluster validity metrics: Two core quantitative indicators were adopted to evaluate clustering reliability: Modularity Q value and Weighted Mean Silhouette S value.

Figure 6 B

Burst detection algorithm: Kleinberg’s burst detection algorithm.

Burst detection parameters: Default parameter configuration was adopted, with the tuning coefficient γ = 0.5 and a minimum burst duration of 1 year to balance detection sensitivity and statistical stability.

Time range: January 1, 2006 to December 5, 2025, consistent with the unified analysis time span of all bibliometric analyses in this study.

Threshold: All cited references were ranked by their citation burst strength. The top 20 publications with the most prominent and statistically significant citation burst trends were selected for visualization.

Figure 7 AB

Counting method: Full counting method.

Normalization method: Association strength normalization.

Clustering algorithm: VOS clustering algorithm.

Threshold setting: A minimum occurrence threshold of 13 times per keyword was applied to filter low-frequency and trivial keywords.

Figure 7 C

Time range & slicing: The analysis timespan was set from January 1, 2006 to December 5, 2025. The entire period was divided into consecutive 1-year time slices (slice length = 1) for consistent network construction.

Selection criterion & network construction parameters: The g-index algorithm was adopted for keyword node selection with k=5. The auxiliary parameter settings were configured as follows: LRF=3.0, L/N=10, LBY=5, e=1.0. Pathfinder network pruning was implemented to remove redundant edges and optimize network clarity.

Figure 7 D

Software & packages: R software (4.3.1) with two packages for visualization: ComplexHeatmap (v2.16.0) and circlize (v0.4.15).

Data source & preprocessing: The analysis adopted the cleaned keyword dataset consistent with other bibliometric analyses. A total of 5784 deduplicated valid keywords were used to construct the keyword co-occurrence matrix. High-frequency core keywords were screened for heatmap plotting to ensure visualization clarity and analytical pertinence.

Matrix construction rule: A symmetrical keyword co-occurrence matrix was established based on the full counting principle, where each matrix cell value represents the cumulative co-occurrence frequency of two corresponding keywords across all included publications.

Clustering setting: Built-in hierarchical clustering algorithms of ComplexHeatmap were applied to row and column dimensions to automatically cluster keywords with highly consistent co-occurrence patterns, gathering thematically similar research topics into identical clusters.

Color mapping setting: The color gradient scheme was generated via the circlize package. Continuous color mapping was adopted to correspond to co-occurrence frequency values, where lighter colors represent lower co-occurrence intensity and darker colors represent higher co-occurrence correlation between paired keywords.

Threshold & screening criteria: Consistent with the keyword co-occurrence analysis standard in this study, only high-frequency keywords that met the unified screening standard were retained for heatmap visualization, eliminating low-frequency trivial keywords that might interfere with clustering and correlation interpretation.

Figure 7 E

Burst detection algorithm: Kleinberg’s burst detection algorithm.

Burst detection parameters: Default parameter configuration was adopted, with the tuning coefficient γ = 0.5 and a minimum burst duration of 1 year, ensuring stable and sensitive identification of valid keyword burst events.

Time range: January 1, 2006 to December 5, 2025, consistent with the unified analysis time span of all bibliometric analyses in this study.

Threshold: All extracted keywords were ranked by their burst strength value. The top 10 keywords with the most significant and statistically robust burst trends were screened and visualized in this figure.

Figure 8 A

Counting method: Full counting method, which performs unbiased statistical counting of gene co-occurrence frequency to guarantee the authenticity and reliability of gene correlation relationships.

Normalization method: Association strength normalization, which standardizes the co-occurrence link strength between genes and eliminates statistical bias caused by unbalanced occurrence frequency of different genes.

Clustering algorithm: Default VOS clustering algorithm of VOSviewer, which aggregates genes with close co-occurrence and functional correlation into independent clusters to identify distinct gene groupings and core research modules.

Threshold setting: A minimum occurrence threshold of 37 times per gene was set to filter low-frequency genes and retain core functional genes. Only genes meeting the minimum occurrence criterion were included in the final visualization to optimize network clarity and highlight pivotal gene correlations.

Figure 8 BC

Software & R packages: R software (4.3.1) with three packages: clusterProfiler, enrichplot, and ggplot2.

Enrichment analysis criteria: Standard hypergeometric distribution test was applied for GO enrichment screening. Statistical thresholds were set as p < 0.05 and adjusted p (FDR) < 0.05 to identify statistically significantly enriched GO terms.

Analysis & visualization workflow: The clusterProfiler package was used to perform GO functional enrichment calculation and gene set annotation; the enrichplot package was adopted to optimize enrichment result sorting, term classification and network layout; the ggplot2 package was utilized for standardized high-quality visualization of enrichment results, including classification distribution and significance ranking of enriched GO terms.

Figure 8 D

Online database & parameter settings: The candidate proteins were imported into the STRING database for interaction network prediction. The species was set as Homo sapiens, and a high confidence score threshold of 0.700 was adopted to filter reliable protein–protein interaction relationships and exclude low-confidence false-positive interactions.

Visualization & analysis software: Cytoscape software was used for secondary visualization, network optimization, and topological parameter calculation of the raw PPI network data exported from the STRING database.

Topological analysis rule: The core topological index degree was calculated for all network nodes using Cytoscape’s built-in analytical tool. Nodes were ranked according to their degree values in ascending order to identify hub proteins with high connectivity and construct the optimized core protein PPI network.

Figure 8 EF

Counting method: Full counting method.

Normalization method: Association strength normalization.

Clustering algorithm: Default VOS clustering algorithm of VOSviewer.

Threshold setting: A minimum occurrence threshold of 40 times per disease was set to filter low-frequency trivial disease terms and retain core diseases closely related to the research. Only diseases meeting the minimum occurrence criterion were included in the final heatmap visualization.

**Definition**

Total link strength (used in VOSviewer): For a given node (e.g., a keyword, author, or country), the total link strength is the sum of the weights of all its links to other nodes.

Burst strength (used in CiteSpace): Burst detection identifies keywords or references that experience a sudden and sharp increase in frequency over a short period. The burst strength value quantifies the intensity of this surge; a higher burst strength indicates a more pronounced spike of attention and thus a stronger emergence of a research frontier theme.

Average citation count: Calculated as the total number of citations received by a set of publications divided by the number of publications in that set.

Modularity Q (network clustering metric): A measure of the quality of network partitioning into clusters. A Modularity Q value typically ranges from 0 to 1. Values greater than 0.3 indicate that the network has a well-defined community structure and the clustering result is significant.

Silhouette S (cluster homogeneity metric): A measure of how tightly grouped and distinct a cluster is. Silhouette scores range from –1 to 1. Values above 0.5 indicate reasonable cluster separation, above 0.7 suggest that the cluster is highly homogeneous, and values close to 1 represent near-perfect consistency within the cluster.
