# Supplementary material for: Bibliometric and visualization analysis of arginine deiminase research from 2006 to 2025: trends, collaboration networks and emerging frontiers
Source: Front Immunol. 2026 Jul 22;17:1784910. doi: 10.3389/fimmu.2026.1784910 (PMC13437694; doi:10.3389/fimmu.2026.1784910)
Supplement: Supplementary file 1 [file SupplementaryFile1.zip › Supplementary Data/Search strategy.docx]

WOS: TS=("padi1" or "padi2" or "padi3" or "padi4" or "padi6" or "*Arginine Deiminase*")

PubMed: ("Protein-Arginine Deiminase Type 1"[Mesh] OR "Protein-Arginine Deiminase Type 2"[Mesh] OR "Protein-Arginine Deiminase Type 3"[Mesh] OR "Protein-Arginine Deiminase Type 4"[Mesh] OR "Protein-Arginine Deiminase Type 6"[Mesh] OR "padi1"[Title/Abstract] OR "padi2"[Title/Abstract] OR "padi3"[Title/Abstract] OR "padi4"[Title/Abstract] OR "padi6"[Title/Abstract] OR "Arginine Deiminase"[Title/Abstract])
